# NK Cell Receptor NKp46 Regulates Graft-versus-Host Disease

**DOI:** 10.1016/j.celrep.2014.05.011

**Published:** 2014-05-29

**Authors:** Hormas Ghadially, Meir Ohana, Moran Elboim, Roi Gazit, Chamutal Gur, Arnon Nagler, Ofer Mandelboim

**Affiliations:** 1 The Lautenberg Center for General and Tumor Immunology, The Hebrew University Hadassah Medical School, IMRIC, Jerusalem 91120, Israel; 2 Hematology Division, Chaim Sheba Medical Center, Tel Hashomer 52621, Israel

## Abstract

Hematopoietic stem cell transplantation (HSCT) is often the only curative treatment for a wide variety of hematologic malignancies. Donor selection in these diseases is crucial, given that transplanted cells can mediate not only the desired graft-versus-leukemia effect but also graft-versus-host disease (GVHD). Here, we demonstrate that in the absence of NKp46, a major killer receptor expressed by human and mouse natural killer (NK) cells, GVHD is greatly exacerbated, resulting in rapid mortality of the transplanted animals because of infection with commensal bacteria. Furthermore, we demonstrate that the exacerbated GVHD is the result of an altered ability of immune cells to respond to stimulation by immature dendritic cells. Because high and low expression of NKp46 on NK cells is observed in different individuals, our data indicate that choosing NKp46-high donors for the treatment of different hematologic malignancies might lead to better tumor eradication while minimizing GVHD.

## INTRODUCTION

Hematopoietic stem cell transplantation (HSCT) is often the only curative treatment for a wide variety of hematologic malignancies. However, the transplanted cells mediate not only the desired graft-versus-leukemia (GVL) effect but also graft-versus-host disease (GVHD), which occurs in up to 80% of patients in mismatch settings (Pérez-Simón et al., 2006). It is therefore essential to select the most proper donor for HSCT, and in this regard it is important that the donor’s major histocompatibility complex (MHC) proteins will be as identical as possible to the patient’s. However, in many cases, it is impossible to achieve a complete identity of the MHC class I proteins, and under these conditions, allogeneic HSCT is considered, which of course increases the chance of GVHD. Therefore, one of the biggest challenges of allogeneic HSCT is preventing GVHD while retaining the beneficial GVL effect.

Natural killer (NK) cells mediate a strong GVL effect but also play an important role in the rejection of allogeneic transplants (Ruggeri et al., 2006) and GVHD (Olson et al., 2010; Shlomchik et al., 1999).

The activity of NK cells is tightly controlled by a balance of signals delivered by activating and inhibitory receptors, which mostly bind to MHC class I proteins. The absence of MHC class I molecules or mismatches between the MHC class I proteins and their cognate NK inhibitory receptors can therefore trigger NK cell activity (Jonsson and Yokoyama, 2009), as long as the target cells expresses activating ligands (Ruggeri et al., 2002). NKp46 is an NK cell killer receptor uniquely expressed by NK cells and a small subset of innate lymphoid cells (Luci et al., 2009; Sanos et al., 2009). Although NKp46 has been shown to be involved in the killing of various tumor cell lines in vitro (Biassoni et al., 1999; Sivori et al., 1999, 2000a, 2000b; Weiss et al., 2004) and in vivo (Gazit et al., 2006; Halfteck et al., 2009), no cellular ligand has been identified so far for this receptor.

Human NK cells are also involved in shaping adoptive immunity, as they can induce maturation of dendritic cells (DCs) through the secretion of tumor necrosis factor α and interferon-γ (IFN-γ), which are triggered by engagement of NKp30 (Vitale et al., 2005), and, on the other hand, DCs are also able to activate NK cells both in vitro (Koka et al., 2004) and in vivo (Lucas et al., 2007). Surprisingly, in-vitro-activated human NK cells have been shown to kill immature monocyte-derived DCs in a natural cytotoxicity receptor (NCR)-dependent manner (Ferlazzo et al., 2002; Spaggiari et al., 2001). In contrast, mature DCs are protected from killing, partly because of the upregulation of MHC class I molecules during maturation (Ferlazzo et al., 2001).

Here, we show that GVHD is exacerbated in the absence of NKp46 (Ncr1 in mice), resulting in increased tissue damage and death due to commensal bacteria infection. This is due to an altered ability of immune cells to respond to stimulation by DCs.

## RESULTS

### Absence of Ncr1 Exacerbates GVHD

Immature dendritic cells (imDCs) are indispensable for the induction of GVHD (Shlomchik, 2007). Since we have recently shown that NKp46 is involved in the killing of imDCs and that in the absence of NKp46 (Ncr1) differences in the T cell responses are observed (Ghadially et al., 2013), we hypothesized that NKp46 could be involved in GVHD.

**Figure 1:**
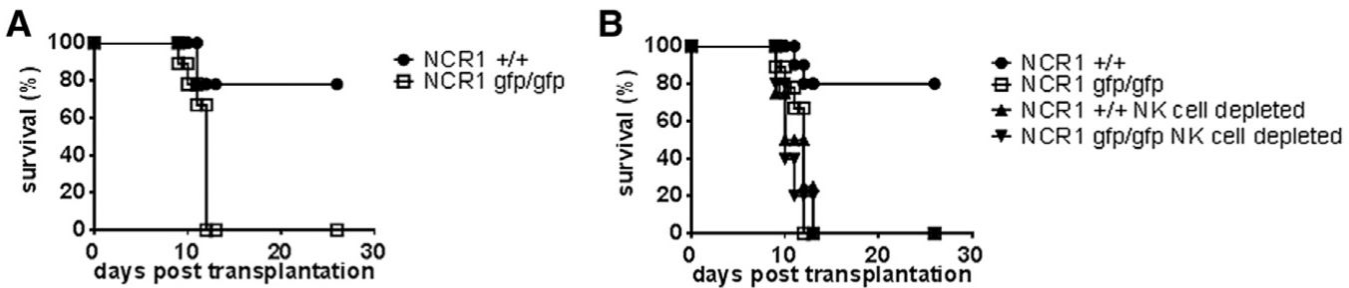
Absence of NKp46 Exacerbates Semiallogeneic GVHD (A) (C57BL/6 × BALB/c) F_1_ mice were injected with 2 × 10^7^ splenocytes from *Ncr1^+/+^* and *Ncr1^gfp/gfp^* mice, and survival was monitored. Shown is one representative experiment out of three performed with nine recipient mice per group. (B) Depletion of NK cells leads to accelerated death after splenocyte transfer. NK cells were depleted from splenocytes before transfer with complement-mediated lysis and anti-NK1.1. Shown is one representative experiment out of two performed with six recipient mice per group.

To study this, we chose a semiallogeneic setting in which splenocytes from wild-type C57BL/6 and Ncr1-deficient mice (*Ncr1^gfp/gfp^*) were injected into (C57BL/6 × BALB/c) F_1_ mice. We used C57BL/6 × BALB/c F_1_ mice because previous experiments had shown that this leads to a relatively mild course of GVHD (Ilan et al., 2007) and thus differences in the strength of the reaction would be easier to identify. As expected, when splenocytes from wild-type mice were injected, the sublethally irradiated recipient mice developed mild signs of GVHD over a period of about 26 days (Figure 1A). Strikingly, when (C57BL/6 × BALB/c) F_1_ mice were injected with splenocytes from *Ncr1^gfp/gfp^* mice, defecation of soft stool and a hunched posture was observed and all mice died within 9 to 12 days. When mice received purified T cells, no differences between the two groups were observed (Figure S1). The rapid mortality was not due to the effect of the irradiation, since mice that had been irradiated but had not received any transplant survived beyond day 56 (data not shown). To demonstrate that this difference in the survival of transplanted mice was indeed due to differences in NK cell function, NK cells were depleted from splenocytes prior to transplantation. Importantly, in the absence of NK cells, mice that had received splenocytes from wild-type as well as from *Ncr1^gfp/gfp^* mice died rapidly between 10 and 12 days posttransplantation (Figure 1B).

**Figure 2:**
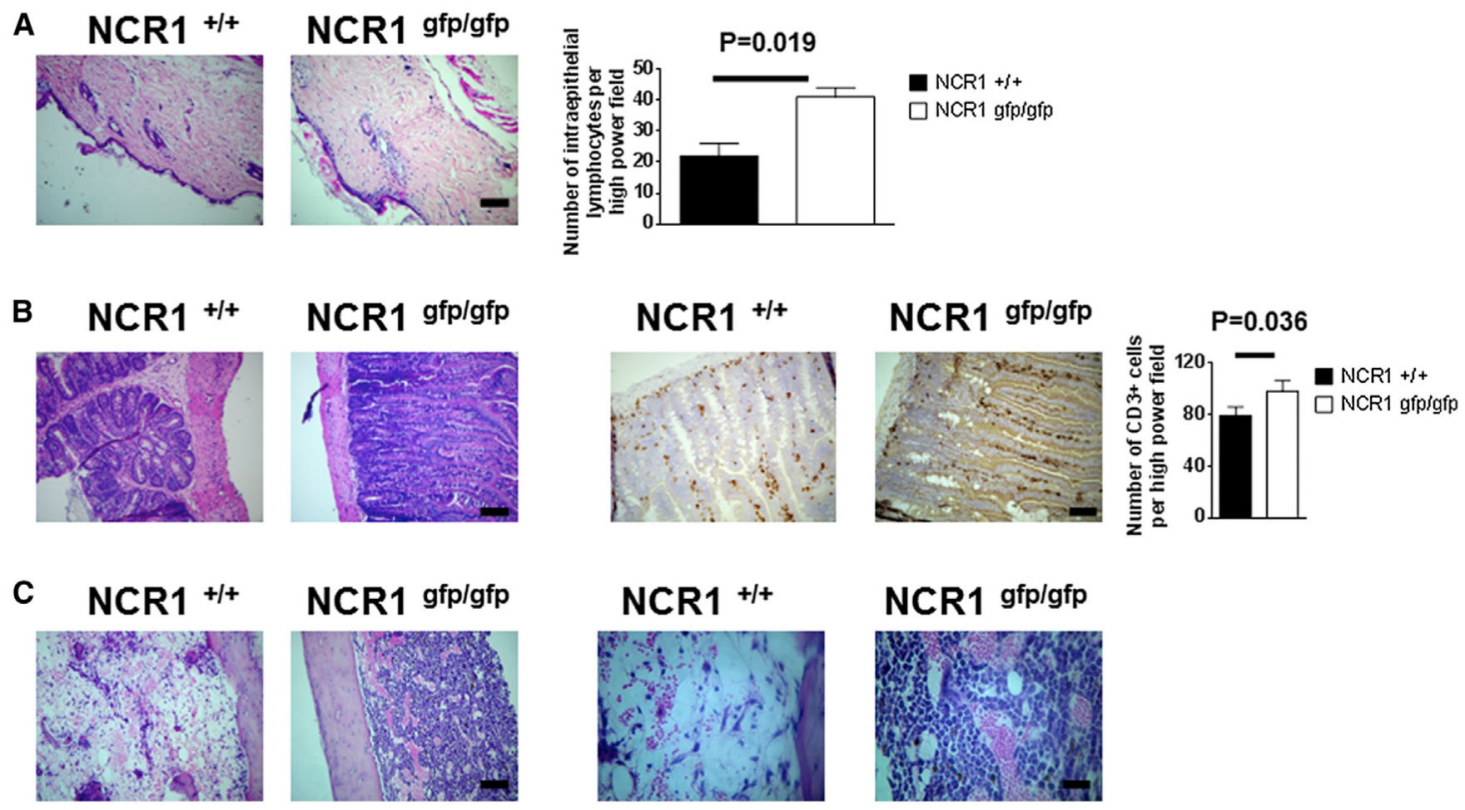
Exacerbated Semiallogeneic GVHD in the Absence of NKp46 (A) Hematoxylin and eosin (H&E) staining of paraffin sections of the skin of F1 mice transplanted with *Ncr1^+/+^* and *Ncr1^gfp/gfp^* splenocytes, 10 days posttransplantation. Original magnification ×10; scale bar represents 10 μm. The right graph shows number of subepidermal lymphocytes per high-power filed. Five fields were randomly selected and counted. Shown are mean values of five fields per section from six different mice from two independent experiments (±SD). Statistical analysis was performed with a two-tailed Student’s t test. (B) Left: H&E staining of paraffin sections of the small intestine of F1 mice transplanted with *Ncr1^+/+^* and *Ncr1^gfp/gfp^* splenocytes, 10 days posttransplantation. Right: immunohistochemical staining of CD3^+^-expressing cells. Original magnification ×100; scale bar represents 1 μm. The right graph shows number of subepidermal lymphocytes per high-power filed. Five fields were randomly selected and counted. Shown are mean values of five fields per section from eight different mice from two independent experiments (±SD). Statistical analysis was performed with a two-tailed Student’s t test. (C) H&E staining of paraffin sections of the bone marrow of F1 mice transplanted with *Ncr1^+/+^* and *Ncr1^gfp/gfp^* splenocytes, 10 days posttransplantation. Left panel is at a ×100 magnification; scale bar represents 1 μm. Right panel is at a ×200 magnification; scale bar represents 0.5 μm.

To find the cause of death of the mice transplanted with *Ncr1^gfp/gfp^* splenocytes, vital organs were recovered and analyzed histologically by staining of sections with hematoxylin and eosin. No pathological alterations were obvious in the lung, brain, heart, kidney, and large intestine (data not shown). Although the liver of transplanted mice showed mild signs of GVHD, no differences between organs of mice transplanted with *Ncr1^gfp/gfp^* and wild-type splenocytes were found on day 9 posttransplantation (Figure S2). Importantly, significantly stronger signs of GVHD were found in the skin of mice transplanted with *Ncr1^gfp/gfp^* splenocytes, characterized by an approximately 50% increase in the number of subepidermal lymphocytes (Figure 2A). Moreover, a significantly higher amount of intraepithelial CD3^+^ lymphocytes was observed in the small intestine in these animals (Figure 2B), a higher proportion of which expressed IFN-γ (Figure S3), suggesting a significantly stronger GVHD in mice transplanted with splenocytes from NCR1^gfp/gfp^ mice than from wild-type mice.

Additionally, femurs and bone marrow of transplanted mice were examined at day 9 posttransplantation (Figure 2C). While the femurs of mice transplanted with wild-type splenocytes contained relatively few cells of different hematopoietic lineages at different stages of maturation, as is normal for regenerating bone marrow following full-body irradiation and transplantation, the marrow of mice transplanted with *Ncr1^gfp/gfp^* splenocytes contained unusually large amounts of cells resembling immature neutrophils. Since this “shift to the left” is indicative of an acute infection, other parameters such as body temperature and white blood cell counts were examined. At day 9, when mice transplanted with *Ncr1^gfp/gfp^* splenocytes started to die, the body temperature dropped to under 32 °C, while it stayed constant in mice transplanted with wild-type splenocytes over the course of the experiment (Figure S4). Additionally, the number of white blood cells in the peripheral blood of these mice was more than ten times higher than in mice transplanted with wild-type splenocytes (1.56 × 10^8^ versus 1.51 × 10^7^ cells/ml).

### Increased Inflammation in the Absence of Ncr1

**Figure 3:**
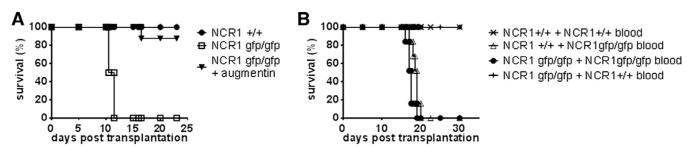
Exacerbated GVHD in the Absence of NKp46 Leads to Sepsis (A) Treatment with antibiotics rescues mice. After transfer of splenocytes, mice were treated with 25 μl of 80 mg/ml Augmentin solution per os every 12 hours over the course of the experiment. Shown is one representative experiment out of two performed with eight recipient mice per group. (B) Transfer of blood from *Ncr1^gfp/gfp^* splenocyte-transplanted mice to nonirradiated *Ncr1^+/+^* and *Ncr1^gfp/gfp^* mice leads to death. (C57BL/6 × BALB/c) F_1_ mice were injected with 2 × 10^7^ splenocytes from *Ncr1^+/+^* and *Ncr1^gfp/gfp^* mice, respectively, and at day 9 posttransplantation, 100 μl blood from each group was injected i.v. into nonirradiated *Ncr1^+/+^* and *Ncr1^gfp/gfp^* mice, respectively. Shown is one representative experiment out of two performed with five recipient mice per group.

As all the above mention pathologies are strong indications of an acute infection, mice were transplanted with splenocytes from wild-type and *Ncr1^gfp/gfp^* splenocytes as before and treated with the broad-spectrum antibiotic Augmentin (0.2 mg/g body weight per day) over the course of the experiment (Figure 3A). Treatment with the antibiotic resulted in a significant increase in the survival of mice that had received *Ncr1^gfp/gfp^* splenocytes. While mice that had received *Ncr1^gfp/gfp^* splenocytes died within 10 to 11 days without treatment, 88% of the mice that had received antibiotics survived for more than 23 days, indicating that the main cause of early mortality is indeed an infection. Treatment with the broad-spectrum antibiotic was not effective when the treatment started 3 days after transplantation (data not shown). To further test this possibility, on day 9 posttransplantation, blood from the various animals was collected and transferred intravenously (i.v.) into nonirradiated C57BL/6 wild-type or *Ncr1^gfp/gfp^* mice. As can be seen in Figure 3B, mice that had received blood from mice that had been transplanted with wild-type splenocytes survived for the course of the experiments and did not show any obvious signs of illness. However, mice that had received blood from animals that had been transplanted with *Ncr1^gfp/gfp^* splenocytes died between day 16 and 21 posttransplantation, irrespective of whether they expressed Ncr1 or not. This suggests that the cause of death of mice transplanted with *Ncr1^gfp/gfp^* splenocytes is indeed an acute infection. Moreover, in the blood of mice that had received *Ncr1^gfp/gfp^* splenocytes, we found *Citrobacter koseri* (Figure S5), which was absent from samples taken from mice transplanted with wild-type splenocytes, while no bacteria was detectable in the blood of mice that had received wild-type splenocytes. Indeed, when infected i.v. with a clinical isolate of *C. koseri*, mice died rapidly, regardless of whether they expressed Ncr1 or not (data not shown). This indicates that the critical step in the sepsis of mice that had received *Ncr1^gfp/gfp^* splenocytes is the infection with commensal bacteria and not the ability to control such an infection.

### Increased Proliferation of Splenocytes from *Ncr1*^gfp/gfp^ Mice after Stimulation with imBM-DCs

**Figure 4:**
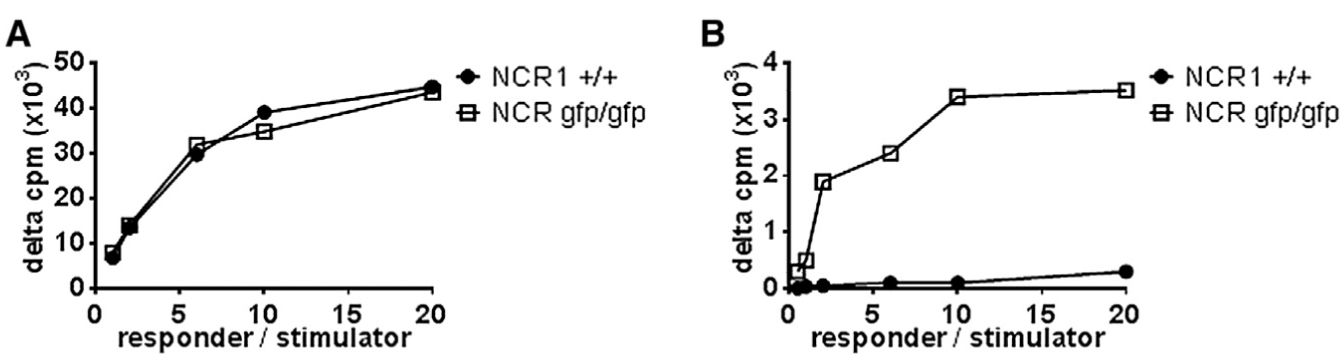
The Ability of Splenocytes to React to Allogeneic Stimulation Is Impaired in the Absence of NKp46 and Exacerbates GVHD Mixed-lymphocyte reactions with splenocytes from wild-type C57BL/6 and *Ncr1^gfp/gfp^* mice as responder cells. Mature lipopolysaccharide-stimulated (1 μg/ml) BM-DCs (A) or immature BM-DCs (B) from BALB/c mice were used as stimulators. Results are expressed as delta cpm (allogeneic-spontaneous) of [^3^H]thymidine uptake. Shown are mean values of three independent experiments performed in triplicate (±SD). *p = 0.005 (two-tailed Student’s t test).

ImDCs are critical for the induction of GVHD (Shlomchik, 2007). We showed recently that killing of imDCs by NK cells is dependent on NKp46 but NKp46 is not involved in the direct interaction of NK cells with T cells (Ghadially et al., 2013), and we reasoned that the absence of NKp46/NCR1 could also have an influence on T cell stimulation in vitro. Therefore, proliferation assays were performed in which we initially incubated mature bone marrow DCs from BALB/c mice as stimulators and splenocytes from wild-type and *Ncr1^gfp/gfp^* mice, respectively, as responder, and as expected, no differences were observed (Figure 4A). In contrast, splenocytes from *Ncr1^gfp/gfp^* mice reacted significantly stronger to stimulation with immature bone marrow dendritic cells (imBM-DCs) (Figure 4B) as compared with the proliferative response of the wild-type splenocytes. Similar results were obtained when splenocytes from (C57BL/6 × BALB/c) F_1_ were used as responder cells (data not shown). ImDCs are known to be poor stimulators of T cells, and indeed the proliferative response of the *Ncr1^gfp/gfp^* splenocytes in reaction to stimulation by imBM-DCs was considerably weaker than the proliferation observed in response to fully mature bone marrow DCs (compare the y axes of Figures 4A and 4B).

## DISCUSSION

Dendritic cells have been shown to be indispensable for the induction of GVHD (Shlomchik, 2007), and it was also shown that donor-derived NK cells kill recipient DCs in the absence of the appropriate inhibitory receptor (Ruggeri et al., 2002). Furthermore, in an allogeneic model of solid organ transplantation, it was shown that host NK cells can kill donor-derived antigen-presenting cells and that this leads to increased rejection of the graft (Yu et al., 2006). Here we used a model of graft-versus-host disease to investigate if the absence of NKp46 would have any effect on the severity of the graft-versus-host reaction. Unexpectedly, mice transplanted with *Ncr1^gfp/gfp^* splenocytes died rapidly in an Ncr1-dependent process mediated by NK cells, since depletion of NK cells prior to transplantation had a similar effect. This rapid death was due to sepsis, since severe hypothermia, increased white blood cell counts, and a massive shift to the left in the bone marrow of these mice was observed. Importantly, treatment of these animals with a broad-spectrum antibiotic resulted in rescue from mortality. Furthermore, transfer of blood from mice that had been transplanted with *Ncr1^gfp/gfp^*, but not wild-type splenocytes, to nonirradiated mice lead to death in these hosts, regardless of their genotype.

But what caused this sepsis? Histological analysis revealed that tissue damage caused by GVHD was more pronounced in the skin and the small intestine of mice that had received *Ncr1^gfp/gfp^* cells. Moreover, we were able to identify the presence of *C. koseri* in the blood of these mice, but not in those transplanted with wild-type splenocytes. *Citrobacter* spp. are gramnegative commensal bacteria that can cause serious infections in immunocompromised hosts, and infection with *C. rodentium* in mice leads to overt symptoms such as defecation of soft stool and a hunched posture (Vallance et al., 2003), which is similar to what was observed in our experiments. When mice were infected i.v. with *C. koseri*, all mice died and no differences between wild-type and *Ncr1^gfp/gfp^* mice were observed. This suggests that the differences observed in the mortality of mice transplanted with wild-type and *Ncr1^gfp/gfp^* splenocytes are not due to a difference in the ability of these animals to control the infection but rather due to whether or not the pathogen reaches the bloodstream. Importantly, and supporting these observations, it has been shown that NKp46 is not involved in the defense against infection by *C. rodentium* (Satoh-Takayama et al., 2009). We therefore propose that the absence of NKp46 on donor NK cells leads to increased stimulation of donor T cells by imDCs that express unknown ligand(s) of NKp46 (Ghadially et al., 2013), which results in exacerbated tissue damage. This tissue damage and the loss of the epithelial barrier function of the small intestine leads to translocation of commensal bacteria into the bloodstream and a rapid death caused by this sepsis.

A recent report (Narni-Mancinelli et al., 2012) suggested that in a mouse in which a mutation of the *Ncr1* gene had been induced by N-ethyl-N-nitrosourea mutagenesis, NKp46-deficient NK cells are hyperresponsive. Substantial differences exist between the mice studied here and the Narni-Mancinelli et al. mice. While the *Ncr1^gfp/gfp^* mice contain a deletion in the *Ncr1* DNA (Gazit et al., 2006), the other mice harbor mutations that preserve the complete *Ncr1* gene (Narni-Mancinelli et al., 2012). Furthermore, while the complete mRNA of *Ncr1* is still transcribed in the other mice (Narni-Mancinelli et al., 2012), in the *Ncr1^gfp/gfp^* mice, it is absent. Finally, at the protein level, the full-length Ncr1 protein is missing in the *Ncr1^gfp/gfp^* mice, while it is retained intracellularly in mice in which the mutation of the *Ncr1* gene had been induced by N-ethyl-N-nitrosourea mutagenesis (Narni-Mancinelli et al., 2012).

Since NKp46 dull and bright phenotypes could be observed in human populations (Sivori et al., 1999), our findings suggest that in cases for which allogeneic transplantation is needed, the preferential donors would be individuals carrying the NKp46-bright phenotype. Furthermore, in humans, NKp30 mediates the killing of immature DCs, and different splice forms of NKp30 have recently been identified that have been shown to influence DC:NK cell crosstalk. Therefore, our data suggest that bone marrow donors could be screened for the presence of the activating NKp30 isoforms to reduce the risk of acute infection.

## EXPERIMENTAL PROCEDURES

### Mice

The *Ncr1^gfp/gfp^* mice were described previously (Gazit et al., 2006). All experiments were done in the specific-pathogen-free unit of the Hebrew University-Hadassah Medical School (Ein Kerem, Jerusalem) according to guidelines of the local ethical Helsinki committee. If not stated otherwise, C57BL/6 mice were used for the experiments.

### Proliferation Assay

Bone marrow DCs were generated as described previously (Lutz et al., 1999). Responder cells of wild-type C57BL/6 or *Ncr1^gfp/gfp^* mice were plated together with lethally irradiated (30 Gy) stimulator cells derived from BALB/c mice (5 × 10^5^ cells/well) at the indicated ratios in a total volume of 0.2 ml medium in triplicate. Cultures were pulsed with 1 mCi/well [^3^H]thymidine (Amersham) on day 4 and harvested 20 hr later. Cells were harvested, and thymidine uptake was quantified in a liquid scintillation counter (TopCount NXT). Results are expressed as delta cpm (allogeneic-spontaneous).

### GVHD

GVHD was induced as described previously (Ilan et al., 2007) with 750 rad of total-body irradiation. This dose has been shown to be nonlethal in C57BL/6 as well as in BALB/c mice. Where indicated, mice were treated with 0.2 mg/g body weight per day with the broad-spectrum antibiotic Augmentin (GlaxoSmithKline) (25 μl of 80 mg/ml per os every 12 hours) for the course of the experiment.

For the transfer experiment, (C57BL/6 × BALB/c) F_1_ were transplanted with splenocytes from *Ncr1^+/+^* and *Ncr1^gfp/gfp^* mice, respectively, as described above. On day 9 posttransplantation, blood was collected and 100 μl from each group was injected i.v. into nonirradiated *Ncr1^+/+^* and *Ncr1^gfp/gfp^* mice, respectively.

### Immunohistochemical Staining

Paraffin-embedded sections were prepared from organs of treated mice. After antigen retrieval with citrate buffer (20 mM) in a pressure cooker (115 °), sections were incubated with a rat CD3 antibody (CD3-12, AbD Serotec) for 2 hr. After repeated washing, sections were incubated with Histofine simple stain MaxPo (Rat; Nichirei Biosciences) for 30 min. Staining was achieved by incubating with 3,3′-diaminobenzidine substrate (DAKO) and counterstaining with hematoxylin.

### In Vitro Complement Lysis

Splenocytes were incubated with 40 μg anti-NK1.1 (PK136, a kind gift from L. Eisenbach) per 1 × 10^6^ cells on ice for 30 min. After washing, the cells were incubated in the presence of 1 ml/1 × 10^8^ cells of rabbit complement (AbD Serotec) for 45 min at 37 °C, during which they were gently agitated every 10 min. After repeated washing steps, cells were counted. Efficiency of depletion, as assessed by flow cytometry with anti-CD49b (DX5), was >97%.

### Statistics

Statistical analysis of the experimental data was performed with a two-tailed Student’s t test. A value of p < 0.05 was considered statistically significant.

## Supplementary Material

Supplementary Information
