# Supplementary material for: NK Cell Receptor NKp46 Regulates Graft-versus-Host Disease
Source: Cell Rep. Author manuscript; Available in PMC 2014 Dec 26. (PMC4074424; doi:10.1016/j.celrep.2014.05.011)
Supplement: Supplementary Information [file NIHMS59194-supplement-Supplementary_Information.pdf]

NKp46 regulates graft-versus-host disease

Hormas Ghadially, Meir Ohana, Moran Elboim, Roi Gazit, Chamutal Gur, Arnon Nagler and Ofer Mandelboim

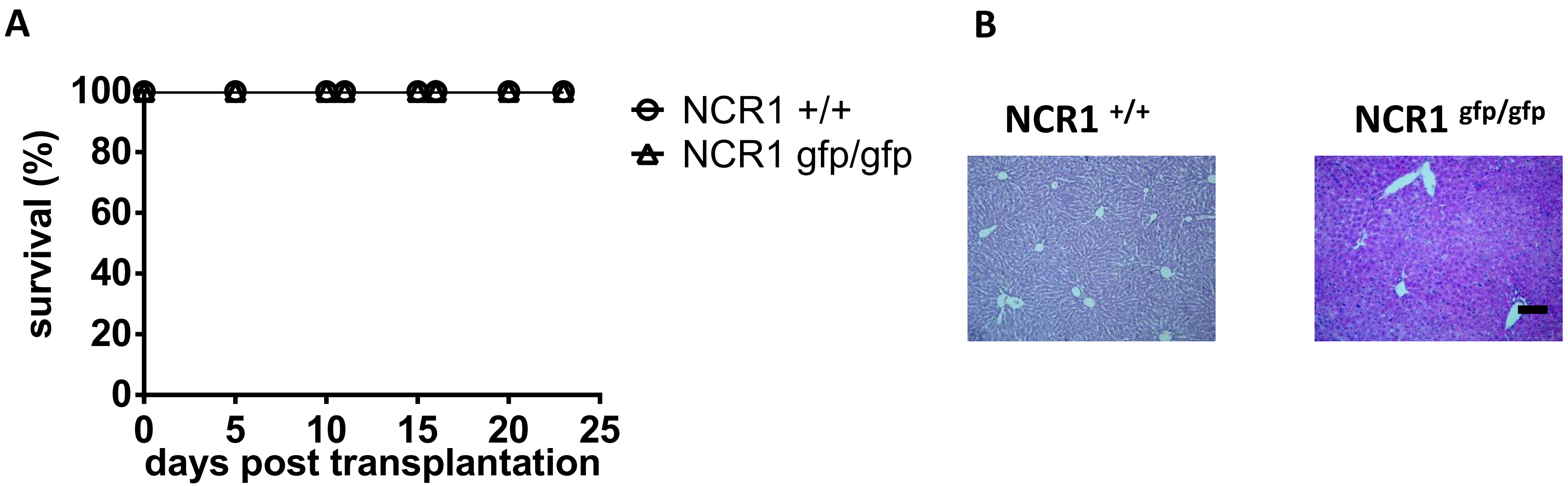

Supplemental Figure S 1. Related to Figure 1.

A: T cells alone do not induce exacerbated semi-allogeneic graft-versus-host disease (GVHD).

T cells were isolated from splenocytes fromNCR1$^{+/+}$ and NCR1$^{gfp/gfp}$ mice, respectively, by magnetic cell separation and (C57BL/6 x BALB/c) F_1 mice were injected with 2×10^7 T cells from and survival was monitored. Shown is one representative experiment out of two performed using four recipient mice per group.

B: No differences in severity of GVHD in the liver.

HE staining of paraffin sections of the liver of (C57BL/6 x BALB/c) F1 mice transplanted with NCR1$^{+/+}$ and NCR1$^{gfp/gfp}$ splenocytes, respectively, nine days post transplantation. Original magnification 10 x, scale bar 10 µm.

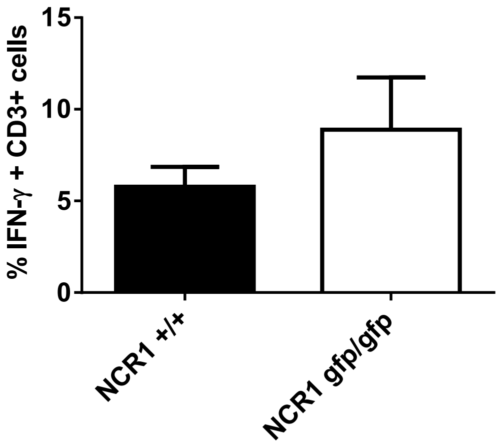

Supplemental Figure S 2. Increased numbers of IFN-g producing cells in the intestine of mice transplanted with splenocytes from NCR1$^{gfp/gfp}$ mice. Related to Figure 2.

(C57BL/6 x BALB/c) F1 mice were injected with 2×10^7 splenocytes from NCR1$^{+/+}$ and NCR1$^{gfp/gfp}$ mice, respectively. On day 10, tissue was harvested, disaggregated and analyzed by flow cytometry for expression of CD3 and intracellular IFN-γ.

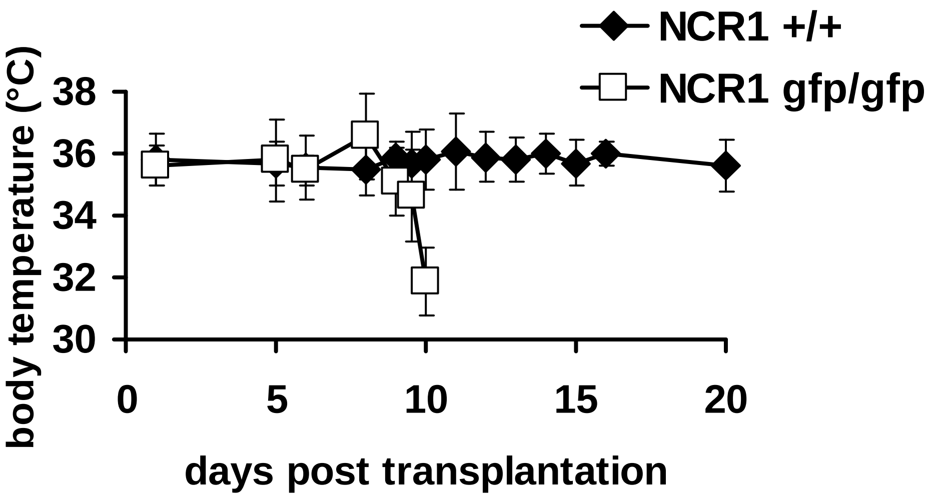

Supplemental Figure S 3. Mice transplanted with NCR1$^{gfp/gfp}$ splenocytes experience hypothermia. Related to Figure 2.

(C57BL/6 x BALB/c) F1 mice were injected with 2 × 10^7 splenocytes from NCR1$^{+/+}$ and NCR1$^{gfp/gfp}$ mice, respectively, and body temperature was measured. Shown is one representative experiment out of three performed using five recipient mice per group.

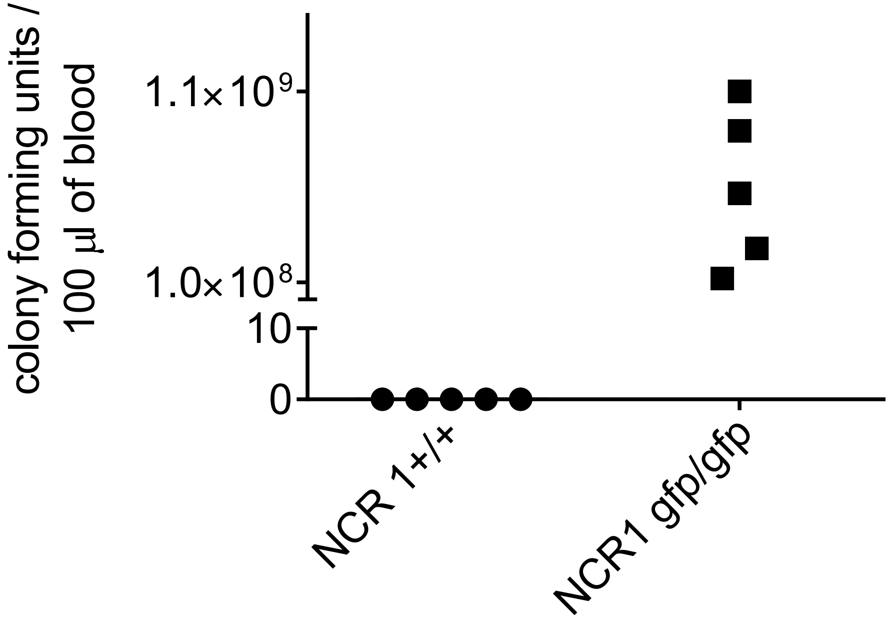

Supplemental Figure S 4. Detection of *Citrobacter koseri* in the blood of transplanted animals. Related to Figure 3.
(C57BL/6 x BALB/c) F1 mice were injected with 2×10^7 splenocytes from NCR1$^{+/+}$ and NCR1$^{gfp/gfp}$ mice, respectively. At day 10 blood was collected by cardiac puncture and analysed for the presence of microorganisms.
